# Untenable Positions with Regard to the Problem of Immunity to Dental Caries*Read before the Liverpool and District Odontological Society, December, 1917. Reprinted from *The Dental Record*, for August, 1905.

**Published:** 1918-09

**Authors:** H. P. Pickerill

**Affiliations:** Professor of Dentistry, Otago University, N. Z.


### UNTENABLE POSITIONS WITH REGARD TO THE PROBLEM OF IMMUNITY TO DENTAL CARIES*

BY MAJOR H. P. PICKERILL, N.Z.M.C.,
Professor of Dentistry, Otago University, N. Z.

Despite the continued use of alkaline dentifrices the incidence of caries increases. Of course, a large proportion of caries occurs in people who never use a dentifrice of any kind, but on the other hand, I do not think it will be claimed by anyone that the incidence of caries is reduced amongst civilized peoples in any inverse proportion whatever to the enormous consumption of alkaline dentifrices taking place all over the world today.

It is interesting to note that the use of alkalies as prophylactics was common for centuries before it was realized that an acid fermentation had any causal relationship to dental caries, and when, after Miller's work, the present chemico-parasitic theory became generally recognized, alkalies were dropped rather in favor of antiseptics—these were not found clinically to do what was hoped and so the pendulum swung back to alkalies—with just a flavoring of antiseptics. The present idea of the use of alkalies is to neutralize the acid formed from fermenting food substances and so prevent the inception of caries. Therein lies a great fallacy.

Lactic acid is formed by the action of organisms upon carbohydrates in the mouth, molecule by molecule slowly and as a nascent acid, and as such it immediately seeks neutralization—it does not and can not accumulate in the mouth and wait until next morning when an overwhelmingly strong alkali is used to neutralize it. It would be most convenient if it did. No, it must be neutralized *at once* either by dentifrice, saliva or lime salts of enamel, and seeing that it is the latter substance which is most

* Read before the Liverpool and District Odontological Society, December, 1917. Reprinted from *The Dental Record*, for August, 1905.

constantly present, the results of such action are most commonly seen.

Miller's observations that lactic acid must reach a certain percentage in the mouth before solution can occur are apt to be most misleading, because it is *not* the mouth as a whole we have to consider, *but a microscopic area of enamel opposite one molecule of lactic acid*, and the question always is, can some diluent or neutralizing agent come along in sufficient time to prevent that microscopic area from being attacked—for once attacked and defeated the total destruction of the tooth, except in comparatively rare cases, is only a matter of time.

It can not be too strongly insisted on that the fight is *at the surface*—the surface once being breached in ninety-nine per cent. of cases total defeat is certain.

The character of many dentifrices too is harmful, in that the particles consist of sharp angular fragments which, with the friction of the toothbrush, considerably abrade the tooth substance. The particles tend to remain at the gingival margin and so form a nidus for the precipitation of salivary salts. Lastly, alkalies in a solid form can never reach those areas of the tooth most commonly attacked, *i. e.*, the apex of the interdental angle and the occlusal fissures.

The most serious disadvantage of alkalies, however, is that not only do they do no good themselves, but they weaken the natural powers of resistance. In other words, they are salivary depressants. In saliva we have a watery fluid containing alkaline salts always available—if called upon—and eminently adapted to neutralize and wash away each molecule of lactic acid as it is formed. It is a potentially perfect mouth wash—no art or device of man could contrive one so beautifully adapted to its purpose. Why then should it not be used? And why, above all, should its use be systematically suppressed by most clumsy and inefficient substitutes?

*Alkalies may be regarded as "resters"* of alimentary excretions. They induce rest of most, if not all, of the alimentary glands—salivary, gastric, pancreatic and hepatic. The results of this are most marked in animals. Animals fed on an alkaline diet firstly do not develop their salivary glands in proportion to the rest of their body, their skins get out of condition and hair falls out, their bones do not ossify as completely as normal—rickets tends to occur. The excretion of calcium and starch goes up above normal. The body weight is markedly self-normal, and finally the animal dies of a polyneuritis. This condition I can produce with certainty in young animals, and I suggest that a similar condition of affairs is going on to a modified extent in young human animals at the present time.

The young human animal has a strong appetite for acid foods—but careful parents invariably eliminate these by practice and precept for fear of subsequent "stomach ache." Instead, children are fed largely on alkali-flavored diets. Bread made with soda, butter adulterated with soda, vegetables boiled in soda, and mineral water made with soda, tea often containing soda—confectionery containing alkaline dyes, and last, but by no means least, dentifrices loaded with alkalies. Have you watched a child cleaning its teeth? He does *not* spit out *all* the dentifrice, especially if it happens to look pretty and is nicely flavored.

The intrinsic result of all this is that we are robbing children of the normal stimulus to their alimentary organs, and the latter robbed of their stimulus fail to develop to their normal strength. The obvious result is carious teeth due to insufficient salivary secretion—"indigestion," due to insufficient gastric secretion—malnutrition and alimentary toxemia and constipation due to insufficient pancreatic, hepatic and intestinal secretions. (Here were shown a number of lantern slides illustrating salivary and alimentary secretion.)

The second idol which I propose to endeavor to shatter is that the enamel of teeth is a "fixed quantity," and varies neither with age nor in different individuals.

This is a tenet of comparatively recent years. It had, I think, until the researches of Miller, Tomes and Black, been generally supposed that there were such things as "hard" and "soft" teeth. Miller and Tomes, however, by a series of chemical analyses came to the conclusion that the enamel of various teeth did not vary. Black, by a series of experiments with an instrument designed to measure resistance to crushing stress, also came to the conclusion that the enamel of teeth did not vary. But the great mistake has been made in drawing the conclusions from these series of investigations that because enamel did not vary in chemical composition or in resistance to crushing force, therefore it did not vary at all! (This is veritably jumping—not to say kangarooing—to conclusions!)

I now propose to show you that the enamel of teeth of different people of various ages *does* vary considerably in important physical properties, besides those of chemical composition and resistance to crushing stress; and I would like to emphasize here, too, again that it is the enamel *surface* which is so important; it is at the very surface of the enamel that the battle is won or lost, the surface having once been breached, the attacks are repeated with increasing severity, as you know from clinical experience, until the whole tooth is destroyed. (Here were shown a number of lantern slides illustrating the variations in hardness, permeability, density and solubility of surface enamel.)

Thus you see, gentlemen, that the enamel of teeth varies as to hardness, as to density, as to permeability and as to solubility—the four most important characteristics when we are considering resistance to caries. It will moreover have been noticed that these variations are all in the same direction, and that is significant. Three things stand out from this series of investigations:

1. That native teeth are harder, more dense, less permeable and less soluble than corresponding European teeth.

2. That the enamel of all teeth tends to become harder, denser, less permeable and less soluble in direct ratio to the number of years after eruption.

3. That the native tooth is no "harder" (using the term in the inclusive sense) prior to eruption than a European tooth.

These, I venture to suggest to you, are most important facts and will have a far-reaching bearing on our campaign against dental caries. Put in other words, the native child and the European start off level with regard to natural resistance of the enamel surface, but the European child is soon left behind in the sclerosing process which goes on in the enamel after eruption; that roughly the native sclerosing power is three times as rapid as that of the European.

That such a sclerosing process is of enormous advantage is incontestable; that the more rapidly it takes place the greater its advantage is also obvious. Is it not therefore worth striving for? Exactly how it occurs would open up another large field for discussion. I believe it occurs through an infiltration (or dialysis) of salivary lime salts through Naysmith's membrane into the uncalcified interprismatic spaces of the superficial strata of enamel; others believe that enamel may receive a lymph supply from the pulp, but at present I think not.

One interesting fact I may note in this connection that as a result of my investigations amongst the less civilized Maoris of New Zealand, I found that the average salivary resistance, *i. e.*, amount per minute of protective substances was *four* times as great as that of the average European child, and you will remember that the sclerosing rate was three times as great.

The third untenable position I propose to deal with is "that a diet of tough or fibrous substances is beneficial to maxillary development in children."

This is a position of comparatively modern establishment. It is one of those doctrines which have but to be stated to be believed almost without question. Superficially speaking and thinking it would seem obvious that the more exercise an organized apparatus was given the better it would be developed; which as a general principle is true; but again it is necessary to look before we leap—to examine minutely both the "take off" and the landing ground, or we may find ourselves, as I have suggested, in an untenable position.

The fallacy lies in the kind of exercise applied and in the kind of development required. Let us consider firstly, what end result we desire to obtain.

We desire, I think, children to develop good strong maxillary bones, but particularly of sufficient width, and with the teeth arranged regularly in good parabolic curves. What we do not desire are long, narrow jaws with the teeth arranged in V-shaped manner on the high Gothic palates. Yet I think you will agree with me that the latter is becoming a definite type which every year is embracing a larger number of our children. Why? I say because their diet is too tough and too fibrous.

Let us consider primarily the mechanism of mastication.

We have one passive factor, a stationary bone, the maxilla—the teeth of which articulate *outside* those of the mandible; the latter is a movable bone and is the active factor; attached to it are a number of very strong muscles which pull it with enormous force in various directions during the act of mastication, and a considerable percentage of this force is transmitted *via* the teeth to the passive maxilla.

Now I think that those who practice orthodontics are more aware than any other practitioners of any other branch of surgery of the extent to which bone is morphologically altered under various stresses and strains. We know that the shape of a bone depends largely on the stress to which it is subject. I go further and say that the shape of a bone depends on the muscular and other stresses to which it is subject. This is perhaps a subject for discussion and opens up a large and interesting field, which I am afraid there is no time to discuss tonight, but in asking you to accept it as a basis of argument I would point out that it is obviously the basis upon which the "position" is founded which I am endeavoring to prove untenable. That is to say, that *if* fibrous substances *are* beneficial it can only be through the medium of the musculature of the jaws. Let us then consider the principal muscles attached to the mandible and used in mastication and observe their results.

Masseter muscle=outward pull 20 mm. each, or 40 altogether.

Int. Pterygoid=*inward* pull of 60 mm.

Mylohyoid=inward pull of 32 mm.

Temporal=outward pull of 40 mm.

Incision=Anterior post. Masseters, outward. Being lost.

Crushing=Masseters and temporals, outward. Being lost.

Trituration=Pterygoids, inward. Increasing.

Swallowing=Mylohyoids, inward. Increasing.

It has been claimed particularly, I think, by Dr. Sim Wallace that it is the action of the tongue in the mastication of tough and fibrous foods which develops the maxillary bones and dental arches by outward pressure. This is another fallacious "axiom."

What is the action of the tongue in mastication? It is used as a pseudopodium—the tip is used as a finger and twists about the mouth in all directions, guiding food outwards between the teeth. But this action is exactly counterbalanced by the action of the buccinator muscle which presses the food *inwards* between the teeth, therefore the action of the tongue as an expander of the jaws from muscular action is a negligible quantity.

The muscles of facial expression, too, are, I believe, largely responsible for the molding of the maxillary arches, and it is just those muscles around the mouth and nose which, when used, have a backward molding pull on the jaws that are falling into disuse.

Finally, I would instance ethnological evidence which shows, I think, conclusively that native races eat much softer and less fibrous foods than we do, and yet undoubtedly have much better developed maxillary bones and much better formed dental arches.

I formerly stated that the food of civilized communities was getting softer and more pap-like. Having further investigated the matter, I desire to considerably qualify these statements. The mistakes lie in not discriminating between the terms "tough" and "hard." The significance lies in the fact that tough things require triturating, and therefore pterygoid and constricting action, whilst hard things require crushing, and therefore masseter, temporal and expanding action. Our dietaries are, I believe, much less hard than they used to be, but also I believe they are, on the whole, much more tough. This opinion, which has gradually been forced upon me, is based upon the following facts and observations·

(1) All primitive people were and are excellent cooks, for the most part steaming nearly all their food until quite tender. Roasting, grilling, stewing are comparatively modern arts.

(2) The food was and is always carefully prepared by grinding, washing, or sieving, before cooking.

(3) Probably more than half of the inhabitants of the world at the present time—the southern and eastern Asiatics, the Hindus, and the native races of South Africa—live on what even the most civilized individuals would regard as extremely soft food—namely, boiled rice, maize and millet. So also the Scotch Highlanders' staple food, is, or was, porridge, and the Irish peasants' potatoes, neither of which could be regarded as other than soft. Yet all these races have characteristically well-shaped and normally developed jaws and teeth.

(4) In conversation with natives—Melanesian and Polynesian—I have found the information always forthcoming unhesitatingly that a white man's meal requires more chewing than their own.

(5) The following are quotations from replies to inquiries that I have recently made in Fiji and New Hebrides groups:

FIJI.—The staple foods are vegetables. Puddings with cocoanut dressing, made by first roasting taro or bread fruit and then pounding it into a stiff paste, are practically swallowed without chewing.

"On the whole, a native repast is not masticated so much as an English meal, because there is less to chew."

MOTA, NEW HEBRIDES.—"Their (the natives') staple food is cooked yam, it is eaten morning and evening—generally roasted, sometimes boiled, often grated and made into a soft pudding (loko) in the ground oven. Bread fruit is next in utility and is either roasted or converted into a dainty mash called 'lot.' Bread fruit is dried in the off season, when it becomes crisp, like hard biscuit, and is thus eaten (kor) or softened in cocoanut sauce, which is a mixture of young cocoanut flesh and sea water."

"No, I should not think that an Islander's ordinary meal would require more mastication than do our meals."

Sir George Simpson, who visited Hawaii in the early part of last century, states that the natives' staple food was Poi, a sour kind of porridge, which was bolted in enormous masses without mastication.

Professor Marshall, who has just returned from Tahiti, informs me that the ordinary food of the natives certainly does not require so much mastication as does our "civilized" food.

(6) From actual subjective experience of living amongst the less civilized Maoris I am able to corroborate all the above statements. Meat when cook by Maoris, was always extremely tender, and their meals called for little or no trituration. I examined numbers of children with perfect jaws and teeth, yet these same children lived almost entirely on soft cooked kumara and riwai, and occasionally maize. Not only so, but they actually refused to eat the crust of ordinary white bread when offered to them, saying that it was not soft enough.

(7) Very much more meat is being consumed in the present day than ever before by civilized communities—in some cases as much as 240 pounds per head per annum, and I venture to assert, from personal experience, that for the most part, the meat when cooked will be vastly tougher, and hence requires more trituration than would be the case if cooked by primitive peoples. Again, take another staple article of diet, bread. As it is usually cooked now-a-days the crusts are tough and rubber-like, instead of being crisp and hard. Now, the important point is that tough things, especially if incision (as it is) is avoided, call for a lateral grinding movement of the mandible and a development of the pterygoid muscles. This, as has been shown, leads to contraction and not to expansion of the jaws.

Swallowing.—Although not usually considered as part of the masticatory act, is really its terminal phase, and it is important to consider it in this connection, because certain of the muscles attached to the lower jaw take part in the movement. The muscles are those attached to the inner surface of the horizontal ramus of the mandible and certain of the hyoid muscles. Swallowing may be abnormal, both as regards character and frequency, though this may depend somewhat on what is regarded as normal.

The act of swallowing is becoming very abnormal, I believe, in both ways. Primitive and natural races eat far less frequently, and so exercise the swallowing muscles much less than we do at the present time. In many such races only one meal a day is taken, in the majority only two, whereas among higher civilized people five and six meals a day are becoming quite common. Again, the swallowing of small boluses, such as ''manners'' now demand, probably requires more contraction of the constricting muscles than do the large boluses of the primitive peoples.

Spasmodic swallowing efforts of an abnormal and chronic character are common in children affected with adenoids and posterior rhinitis. In these cases the child is constantly making strained efforts to swallow either post-nasal growths or the thick viscous secretion on the posterior pharyngeal wall. The superior constrictor, the mylo-hyoid, and the digastric muscle are therefore frequently and forcibly contracting, and these all produce an inward pull upon the mandible.

We have, therefore, by a process of analysis, elucidated the fact that modern dietetic habits are tending to the hypertrophy of those muscles which contract the jaws, and to the disuse of those muscles which would expand the jaws.

